# Short- and long-term outcomes of totally robotic versus robotic-assisted radical distal gastrectomy for advanced gastric cancer: a mono-institution retrospective study

**DOI:** 10.1186/s12957-019-1722-5

**Published:** 2019-11-11

**Authors:** Rui Luo, Dongning Liu, Shanping Ye, Hechun Tang, Weiquan Zhu, Penghui He, Cheng Tang, Taiyuan Li

**Affiliations:** 1 Medical College of Nanchang University, Nanchang, 330000 China; 2 Department of General Surgery, The First Affiliated Hospital of Nanchang University, Nanchang, 330000 China

**Keywords:** Distal gastric cancer, Robotic surgery, Advanced gastric cancer, Propensity score matching

## Abstract

**Purpose:**

By comparing short- and long-term outcomes following totally robotic radical distal gastrectomy (TRDG) and robotic-assisted radical distal gastrectomy (RADG), we aimed to assess in which modus operandi patients will benefit more.

**Methods:**

From January 2015 to May 2019, we included 332 patients undergone RADG (237) and TRDG (95). Based on the propensity score matching (PSM), inclusion and exclusion criteria, 246 patients were finally included in the propensity score-matched cohort including RADG group (164) and TRDG group (82). We then compared the short- and long-term outcomes following both groups.

**Results:**

Propensity score-matched cohort revealed no significant differences in both groups. Intra-abdominal bleeding, time to pass flatus, postoperative activity time, length of incision hospital stays, and stress response were significantly less in TRDG group than in RADG group. We observed 30 complications in RADG group while 13 complications in TRDG group. There were no significant differences in TRDG group and RADG group in terms of operation time, time for anastomosis, proximal resection, distal resection margin, number of lymph node resection, and total hospitalization cost. Both 3-year overall survival and 3-year disease-free survival were comparable in both groups.

**Conclusions:**

TRDG is a safe and feasible modus operandi profiting from short- and long-term outcomes compared with RADG. As surgeons improving their professional skills, TRDG could serve as the standard procedure for distal locally advanced gastric cancer with D2 lymphadenectomy.

## Introduction

Gastric cancer (GC) is the fifth common clinical gastrointestinal tract tumor worldwide ranking the third in the cause of death from cancer [1]. Specially, radical distal gastrectomy with D2 lymphadenectomy approach is the dominating means to relieve tumor burden in patients with distal locally advanced gastric cancer (AGC) and has gained global consensus to be a standard procedure [2, 3]. With the advent of a robot, minimally invasive surgery has stepped into a new era where patients endure less physical pain and tumor burden compared with traditional open surgery [4, 5]. Currently, da Vinci robotic-assisted system (DRAS) is the most widely employed robotic surgery system in clinical practice developed by Intuitive Surgical Inc. which has developed from the first generation approved for clinical use in 2000 to the fourth generation [6]. Surgical robot has garnered immense preference from general surgeons in minimally invasive surgery for gastric cancer on account of numerous merits compared with laparoscopic surgery including the following: to patients, it allows better anastomosis cosmesis, less physical pain, shorter hospital stays, faster recovery of gastrointestinal function, and less time in recovery of passing flatus, nevertheless guaranteeing considerable oncological safety; to surgeons, it provides a 3D imaging and high definition, enlarges operative fields, filters through physical tremor, and permits multiple-arms operation [4, 6–8].

Regarding radical distal gastrectomy, with the help of DRAS, there are two alternative modus operandi including robotic-assisted distal gastrectomy (RADG) with D2 regional lymphadenectomy and totally robotic distal gastrectomy (TRDG) with D2 regional lymphadenectomy. In spite of the aforementioned advantages, many questions need to be answered and defined, foremostly, which modus operandi will patients benefit from more. However, few literatures have ever reported regarding the short- and long-term outcomes following RADG and TRGD. Hence, to explore further and to work out this issue, in this study, we collected 332 patient cases and used propensity score matching analysis in order to eliminate the bias of each patient assigned to the two different study groups. The purpose of this study was to compare the short- and long-term outcomes following RADG and TRGD to assess in which modus operandi patients will benefit more.

## Materials and methods

### Patient background

In this retrospective clinical study, we gathered and analyzed 332 cases undergone radical distal gastrectomy from a big single surgeon center: The First Affiliated Hospital of Nanchang University from January 2015 to May 2019 including RADG (237) and TRDG (95). All these patients personally signed the consent for surgery. All the patient medical records were extracted from the prospectively maintained database at the Department of Gastrointestinal Surgery in The First Affiliated Hospital of Nanchang University. Our research was approved by the Ethics Committee of The First Affiliated Hospital of Nanchang University.

### Propensity score matching, inclusion, and exclusion criteria

**Fig. 1:**
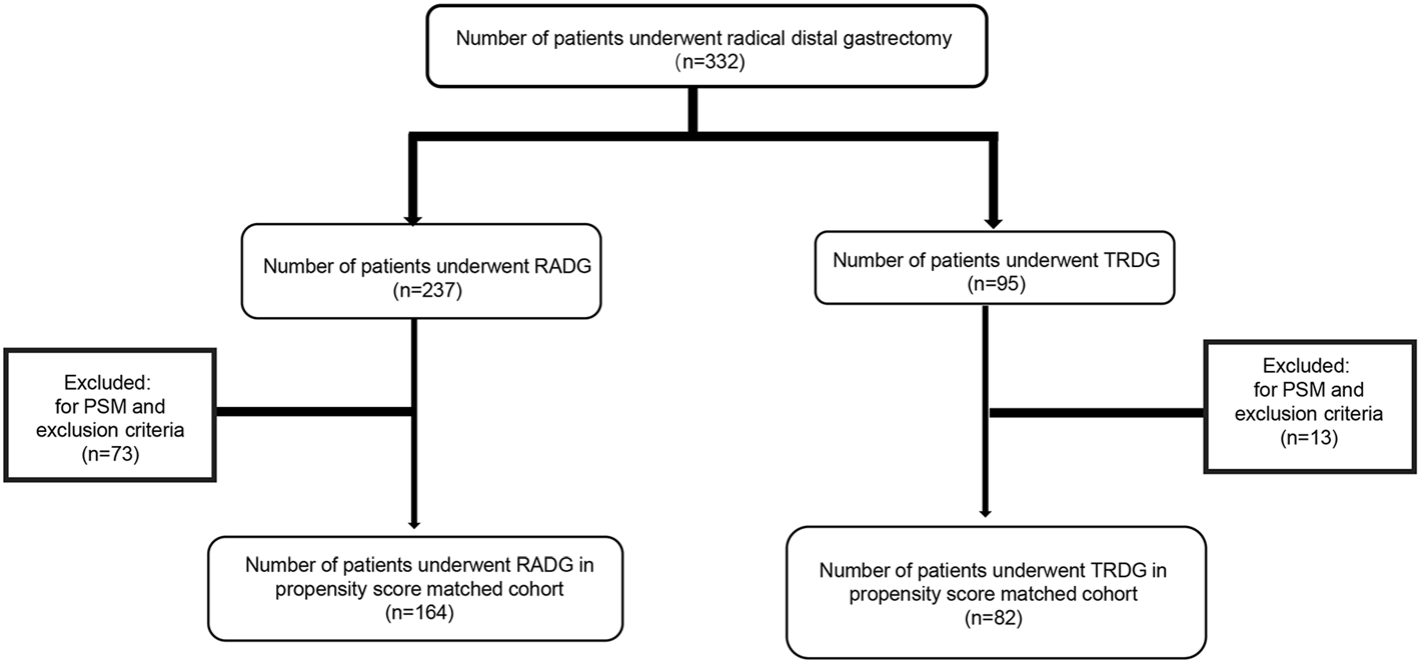
Study profile

Since there is a statistical significant difference (parameter gender) in clinical-pathological characteristics before propensity score matching (PSM) between the two groups in the entire cohort in Table 1, to compensate the bias in each group, we performed a 1:2 ratio PSM based on these following parameters as predictors: gender, age, BMI, American Society Anesthesiologists (ASA) physical status, T stage, N stage, Clinical TNM stage, histology, preoperative s-CEA, preoperative s-CA199, and preoperative s-CA125. After PSM and passing the inclusion and exclusion criteria, 246 patients (Fig. 1) finally formed the propensity score-matched cohort including RADG (164) and TRDG (82). Inclusion and exclusion criteria applied to each group in this study are as follows:

**Table 1:** Clinical-pathological characteristics

	Entire cohort			Propensity score matched cohort		
Parameters	RADG	TRGD	*p*	RADG	TRGD	*p*
	(*n* = 237)	(*n* = 95)		(*n* = 164)	(*n* = 82)	
Gender			0.536			0.778
Male	142 (59.9%)	56 (58.9%)		97 (59.1%)	50 (61.0%)	
Female	95 (40.1%)	39 (41.1%)		67 (40.9%)	32 (39.0%)	
Age, year	58.6 ± 11.0	55.2 ± 11.9	*0.001*	55.8 ± 10.6	55.5 ± 10.3	0.758
BMI, kg/m^2^	22.7 ± 2.6	23.3 ± 2.8	0.457	22.8 ± 2.7	23.1 ± 2.5	0.623
ASA physical status			0.145			0.636
I	85 (35.9%)	31 (32.6%)		52 (31.7%)	23 (28.0%)	
II	122 (51.5%)	52 (54.7%)		88 (53.7%)	49 (59.8%)	
III	30 (12.7%)	12 (12.6%)		24 (14.6%)	10 (12.2%)	
T stage			0.614			0.828
T1	28 (11.8%)	11 (11.6%)		19 (11.6%)	10 (12.2%)	
T2	66 (27.8%)	26 (37.4%)		46 (28.0%)	22 (26.8%)	
T3	89 (37.6%)	33 (34.7%)		65 (39.7%)	32 (39.0%)	
T4a	54 (22.8%)	25 (26.3%)		34 (20.8%)	18 (22.0%)	
*N* stage			0.559			0.814
N0	94 (39.7%)	36 (37.9%)		65 (39.6%)	32 (39.0%)	
N1	71 (30.0%)	30 (31.6%)		49 (29.9%)	24 (29.3%)	
N2	45 (19.0%)	17 (17.9%)		29 (17.7%)	14 (17.1%)	
N3	27 (11.4%)	12 (12.7%)		21 (12.8%)	12 (14.6%)	
Clinical TNM stage			0.468			0.889
I	28 (11.8%)	11 (11.6%)		19 (11.6%)	9 (11.0%)	
II	79 (33.3%)	26 (27.4%)		54 (32.9%)	18 (22.0%)	
III	130 (54.9%)	58 (61.1%)		91 (55.5%)	55 (67.0%)	
Histology			0.755			0.789
Differentiated	182 (76.8%)	72 (75.8%)		123 (75.0%)	63 (76.8%)	
Undifferentiated	55 (23.2)	23 (24.2%)		41 (25%)	19 (23.2%)	
Specimen length^,^ cm	14.6 ± 3.8	14.1 ± 3.2	0.550	14.4 ± 3.6	14.2 ± 3.3	0.592
Preoperative s-CEA, ng/ml	2.6 ± 0.5	2.9 ± 0.6	0.345	2.7 ± 0.5	2.9 ± 0.6	0.355
Preoperative s-CA199, U/ml	14.6 ± 2.6	15.1 ± 2.8	0.789	14.9 ± 2.7	15.2 ± 2.8	0.794
Preoperative s-CA125, U/ml	11.4 ± 2.1	11.9 ± 2.3	0.514	11.6 ± 2.2	11.8 ± 2.3	0.518

Abbreviations: *BMI* body mass index, *ASA* American Society of Anesthesiologists, *CEA* carcinoembryonic antigen, *s-CA199* serum carbohydrate antigen 199, *s-CA125* serum carbohydrate antigen 199

### The inclusion criteria

1. Preoperative gastroscopy and pathological biopsy of all patients were adenocarcinomas
2. Depth of invasion was diagnosed with advanced gastric cancer (AGC) by pathological identification by using AJCC/UICC guidelines (8th,2017)
3. There is no distant metastasis or invasion to adjacent organs and tissues
4. There is no another malignancy
5. There is no preoperative chemotherapy or radiation therapy

### The exclusion criteria

1. Acute, gastric perforation, and so on that need emergency surgery
2. Gastric stump cancer
3. Distant metastasis or intraoperative confirmation of implantation metastasis
4. Nonradical distal dissection with D2 regional lymphadenectomy
5. Palliative surgery

### Surgeon background

All operations in this study were performed by the same chief surgeon (Taiyuan Li), who had performed more than 1500 cases of robotic gastrointestinal surgery since January 2015.

### Statistics and analysis

All statistical analyses were carried out by SPSS (SPSS Inc., version 22.0, Chicago, IL, USA). Continuous variables were expressed as mean ± standard deviation (mean ± SD), and metric variables are expressed as number (*n*). In this study, we used Pearson chi-square test and Mann-Whitney *U* test to calculate the *p* value of each parameter in each table. Survival analysis was processed by the Kaplan-Meier method and log-rank test was used to compare the statistical significance between TRDG and RADG group. Statistical significance was set at *p* < 0.05.

The following parameters were statically analyzed:

1. Clinical–pathological characteristics: gender, age, BMI, ASA physical status, T stage, N stage, clinical TNM stage, histology, specimen length, preoperative s-CEA, preoperative s-CA199, and preoperative s-CA125. T stage, N stage, and clinical TNM stage were classified based on the American Joint Committee on Cancer/Union for International Cancer Control guidelines (AJCC/UICC guidelines, 8th, 2017) for gastric cancer.
2. Short-term outcomes:

- General conditions: operation time, intra-abdominal bleeding, time for anastomosis, proximal resection margin, distal resection margin, number of lymph node dissection, time to pass flatus, postoperative activity time, postoperative activity time, length of incision, hospital stays, and total hospitalization cost.
- Early complications: postoperative gastric paralysis, bowel obstruction, intra-abdominal bleeding, intra-abdominal abscess, pulmonary complications, wound infection, anastomotic bleeding, anastomotic leakage, internal hernia, seroma, and pancreatic fistula.
- Surgical stress response: C-reactive protein (CRP), procalcitonin (PCT), white blood cell (WBC), and interleukin-6 (IL-6). All these parameters were tested preoperatively as a baseline, 1st day after surgery, 3rd day after surgery, and 5th day after surgery.

(3). Long-term outcomes: overall survival and disease-free survival.

### Surgical operative process

#### The same processes

Most operative processes for RADG and TRDG were identical. Following procedures were based on our surgeons experience and the guidelines [9]. All the surgeries were done with the help of the da Vinci Si system.

**Fig. 2:**
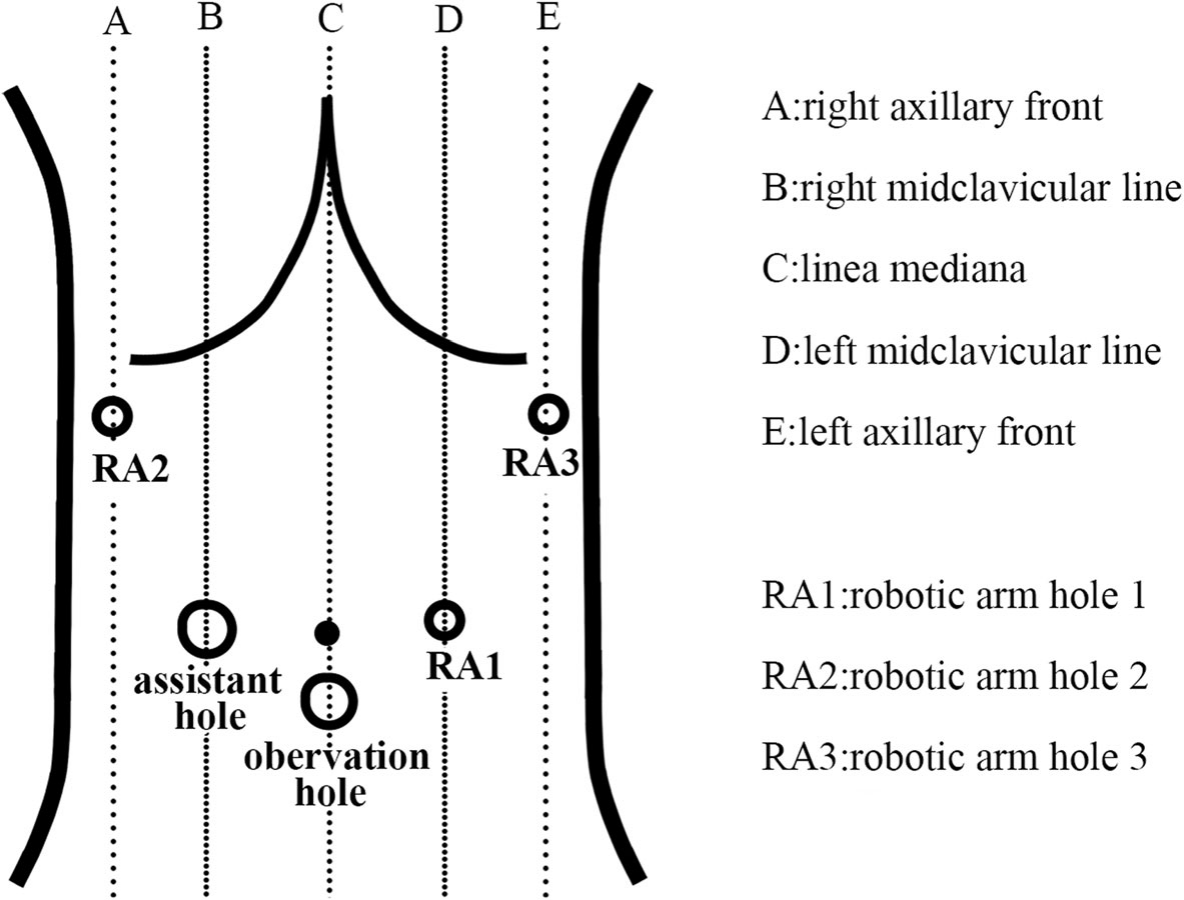
Trocar hole layout

1. Anesthesia and position: The patients were performed endotracheal intubation and general anesthesia laying on his back with his legs apart and his head high with low feet. The long axis of the body should be 15~20° from the horizontal axis.
2. Trocar location: We generally adopted the “U” type 5-hole layout (Fig. 2). Puncture 12 mm Trocar as the observation hole at 1 cm below the umbilicus and pneumoperitoneum hole with pressure 10~12 mmHg. Puncture 8 mm Trocar 1~2 cm below the costal margin of the left axillary front as the 3rd manipulator hole. In the position where left midclavicular line intersects umbilical horizontal line, puncture 8 mm Trocar as the 1st manipulator hole. Puncture 8 mm Trocar 1~2 cm below the costal margin of the right axillary front as the 2nd manipulator hole. Puncture 12 mm Trocar in the position where the right midclavicular line intersects umbilical horizontal line as the assistant hole.
3. Abdominal exploration: Establish pneumoperitoneum (pressure 10~12 mmHg), then use robotic laparoscopy for laparoscopic exploration aiming at assuring whether there was peritoneal effusion, liver, mesangial adhesion, and peritoneal metastasis. Finally, fix the robotic arms after the surgery could be performed explicitly.
4. Connection of robotic surgical system and operator location: The robotic arm system was placed on the side of the head of the patient, right on the center line of the patient’s body. Each arm took the “embrace” posture, with lens arm centered and bilateral properly abducted. Robotic arm 1 was connected to the ultrasonic knife system, robotic arm 2 was connected to the non-damaging gripper, and robotic arm 3 was connected to the electrocoagulation bipolar. The assistant is on the right side.
5. Lymphadenectomy: D2 radical lymphadenectomy was performed in accordance with the guidelines [9].
  - Robotic arms 2 and 3 lifted the transverse mesocolon, and the assistant pulled the transverse colon on the opposite side, robotic arm 1 (ultrasound knife) cut the omentum along the transverse colon and separated the anterior lobe of the transverse mesentery. Then, the lymph nodes of group 4 were firstly dissected.
  - Separate splenic vein, then cut off left gastro-omental vein, gastro-omental artery, and two short gastric vessels by the root, and finally, the lymph nodes of group 6 were dissected from left to right.
  - Cut the hepato-gastric ligament along the liver, group 2 lymph nodes were then cleared above the pylorus.
  - Robotic arms 2 and 3 lifted the stomach and cleared the common lymph nodes around the hepatic artery and celiac trunk (group 8 and 9 lymph nodes) then ligate and cut off the left gastric artery and left gastric vein along their roots and then remove group 7 lymph nodes.
  - Remove fat tissue and lymphatic tissue around hepato-gastric ligament and lesser curvature upward to the cardia. Finally, dissect group 1 and 3 lymph nodes and cut off the spleen-gastric ligament. Transect the duodenum with a liner stapler at 3 cm from the distal end of the anterior pyloric vein.

Hereto, the robotic gastric mobilization was over.

#### The different processes

1. TRDG

- Specimen excision: extend a 60-mm straight liner stapler from the assistant hole and separate the specimen at a point no less than 2 cm at the upper pole of the tumor, then remove about 70% distal stomach together with the size of the omentum and surrounding fatty lymphoid tissue. The assistant placed the specimen separated into the specimen bag, then tightened the suture of the specimen bag and placed it on the lower abdomen.
- Gastrointestinal anastomosis: Lift the jejunum about 20 cm below the trochanteric ligament, then cut a hole about 1 cm in size from the ileal wall and the same method to take an opening from the remnant stomach about 1 cm. Finally, extend 45 mm linear cutting closure from the assistant hole and then insert it between the stomach and jejunum. Finally, gastrointestinal lateral anastomosis was performed, 3.0 barb wire was used to suture the remaining openings. The duodenal stump was strengthened with continuous suture and buried in the string suture. After abdominal lavage and no obvious bleeding, a drainage tube was placed in through robotic armhole 2.
- Specimen removal: Remove the specimen from the incision of about 3 cm made at the observation hole. Finally, seam the entire abdominal wall layer by layer.
  (2) RADG

After the robotic gastric mobilization is over, detach and remove the robotic system and then make an incision about 7 cm at the epigastrium. Insert the protective ring and pull out the dissociative stomach and omentum. Then, perform the specimen excision and Billroth II anastomosis in vitro the same as the “gastrointestinal anastomosis” procedures mentioned in TRDG group. The subsequent surgical procedures are the same as laparotomy [10]

#### Subsequent treatment

To palliate the symptoms and improve survival time and life quality, patients in this study with distal locally advanced gastric cancer were prescribed with fluorouracil-based combination regimens according to the guidelines [11].

## Results

### Operative outcomes for the propensity score-matched cohort and patient clinical-pathological characteristics

Before PSM, there was a significant difference (age) between RADG group and TRDG group (58.6 ± 11.0 year vs. 55.2 ± 11.9 year, *p* = 0.001) in the entire cohort. Age is an important factor affecting survival time regarding long-term outcomes [12]; to balance the difference between the two groups, we performed propensity score matching and excluded some patients according to the exclusion criteria (Fig. 1). Propensity score-matched cohort (Table 1) depicts the clinical pathological outcomes after passing propensity score matching, inclusion, and exclusion criteria. No significant differences could be observed between the two groups in terms of gender, age, BMI, ASA physical status, T stage, N stage, clinical TNM stage, histology, specimen length, preoperative s-CEA, preoperative s-CA199, and preoperative s-CA125.

### Short-term outcomes

All patients underwent surgical operation successfully. Considering general conditions (Table 2), intra-abdominal bleeding in TRDG group was significantly reduced than that in RADG (38.0 ± 18.5 ml vs. 85.6 ± 35.8 ml, *p* = 0. 001) (Table 2). Time to pass flatus, postoperative activity time and hospital stays were all significantly less in TRDG group than in RADG group with respective statistical data and *p* value (2.7 ± 0.9d vs. 3.8 ± 0.8d, *p* = 0.001; 1.2 ± 0.8d vs. 3.3 ± 0.8d, *p* = 0.001; 8.2 ± 1.6d vs. 10.4 ± 2.5d, *p* = 0.001). Interestingly, the length of incision in TRDG was much shorter than in RADG (3.3 ± 1.2 cm vs. 6.8 ± 2.5 cm, *p* = 0.001) with average length falling nearly by half. There were no differences regarding operation time, time for anastomosis, proximal resection margin, distal resection margin, number of lymph node dissection, and total hospitalization cost.

**Table 2:** General conditions

Parameters	RADG	TRDG	*p*
	(*n* = 164)	(*n* = 82)	
Operation time, min	277.1 ± 22.8	282.8 ± 32.8	0.198
Intra-abdominal bleeding, ml	85.6 ± 35.8	38.0 ± 18.5	*0.001*
Time for anastomosis, min	73.4 ± 8.3	74.5 ± 10.2	0.458
Proximal resection margin, cm	6.01 ± 1.03	6.06 ± 1.24	0.784
Distal resection margin, cm	6.05 ± 0.98	6.32 ± 1.21	0.125
Number of lymph node dissection, n	34.6 ± 9.5	33.4 ± 9.7	0.440
Time to pass flatus, days	3.8 ± 0.8	2.7 ± 0.9	*0.001*
Postoperative activity time, days	3.3 ± 0.8	1.2 ± 0.8	*0.001*
Length of incision, cm	6.8 ± 2.5	3.3 ± 1.2	*0.001*
Hospital stays, days	10.4 ± 2.5	8.2 ± 1.6	*0.001*
Total hospitalization cost, $	9011.7	9447.8	0.159

Footnote: Time for anastomosis in TRDG group was calculated from the surgeon operating the robot to the abdominal closure, preoperative disinfection, towel time, and anesthesia is not included. Time for anastomosis in RADG group was calculated from the beginning of transecting duodenum to the end of gastrointestinal anastomosis and detaching the robotic system

Regarding early complications (Table 3) in RADG group, a total of 30 complications (18.3%) were observed including 2 postoperative gastric paralysis, 3 bowel obstruction, 1 intra-abdominal bleeding, 1 intra-abdominal abscess, 10 pulmonary complications, 8 wound infection, 2 anastomotic bleeding, 2 anastomotic leakage, 0 internal hernia, 0 seroma, and 1 pancreatic fistula. To our expectation, 13 complications (15.9%) were observed in TRDG group with respective frequency: 1 postoperative gastric paralysis, 1 bowel obstruction, 1 intra-abdominal bleeding, 0 intra-abdominal abscess, 4 pulmonary complications, 4 wound infection, 0 anastomotic bleeding, 1 anastomotic leakage, 0 internal hernia, 0 seroma, and 1 pancreatic fistula.

**Table 3:** Early complications

Parameter	RADG	TRGD	*p*
	(*n* = 164)	(*n* = 82)	
Total	30 (18.3%)	13 (15.9%)	0.246
Postoperative gastric paralysis	2	1	–
Bowel obstruction	3	1	–
Intra-abdominal bleeding	1	1	–
Intra-abdominal abscess	1	0	–
pulmonary complications	10	4	–
Wound infection	8	4	
Anastomotic bleeding	2	0	–
Anastomotic leakage	2	1	–
Internal hernia	0	0	–
Seroma	0	0	–
Pancreatic fistula	1	1	–

All the indexes to evaluate the surgical stress response in this study were described in Table 4. No significant differences were observed preoperatively in both groups. Nevertheless, compared with RADG group, CRP levels in TRDG group were significantly lower no matter in day 1, day 3, or day 5 (85.3 ± 38 mg/L vs. 40 ± 24.4 mg/L, *p* = 0.029; 89.7 ± 33 mg/L vs. 58.6 ± 19.5 mg/L, *p* = 0.025; 46 ± 16.8 mg/L vs. 21 ± 12.3 mg/L, *p* = 0.019) as well as IL-6 levels (393 ± 191 pg/mL vs. 232 ± 133 pg/mL, *p* = 0.032; 313 ± 151 pg/mL vs. 112 ± 57 pg/mL, *p* = 0.025; 363 ± 181 pg/ml vs. 84 ± 43 pg/ml, *p* = 0.013). No significant differences were observed postoperatively on any given day between TRDG group and RADG group in terms of PCT levels and WBC levels.

**Table 4:** Surgical stress response

Parameters	Preop			Day 1			Day 3			Day 5		
	RADG	TRDG	*p*	RADG	TRDG	*p*	RADG	TRDG	*p*	RADG	TRDG	*p*
CRP (mg/L)	6.3 ± 4.3	5.1 ± 2.3	0.523	85.3 ± 38	40 ± 24.4	*0.029*	89.7 ± 33	58.6 ± 19.5	*0.025*	46 ± 16.8	21 ± 12.3	*0.019*
IL-6 (pg/mL)	68 ± 33	60 ± 29	0.625	393 ± 191	232 ± 133	*0.032*	313 ± 151	112 ± 57	*0.025*	**363 ± 181**	84 ± 43	*0.013*
PCT (ng/mL)	7 ± 3.2	6 ± 3	0.472	6.3 ± 3.1	8 ± 4.4	0.256	6.0 ± 2.8	6.7 ± 3.1	0.486	2.7 ± 1.8	3.4 ± 1.3	0.358
WBC (× 10^9/L)	5.1 ± 1.3	6.4 ± 1.9	0.526	13.2 ± 4	11 ± 2.7	0.348	10.7 ± 3.1	9.1 ± 2.269	0.589	8.2 ± 2.5	6.3 ± 1.5	0.386

*CRP* C-reactive protein, *IL-6* interleukin-6, *PCT* procalcitonin, *WBC* white blood cell

### Long-term outcomes

**Fig. 3:**
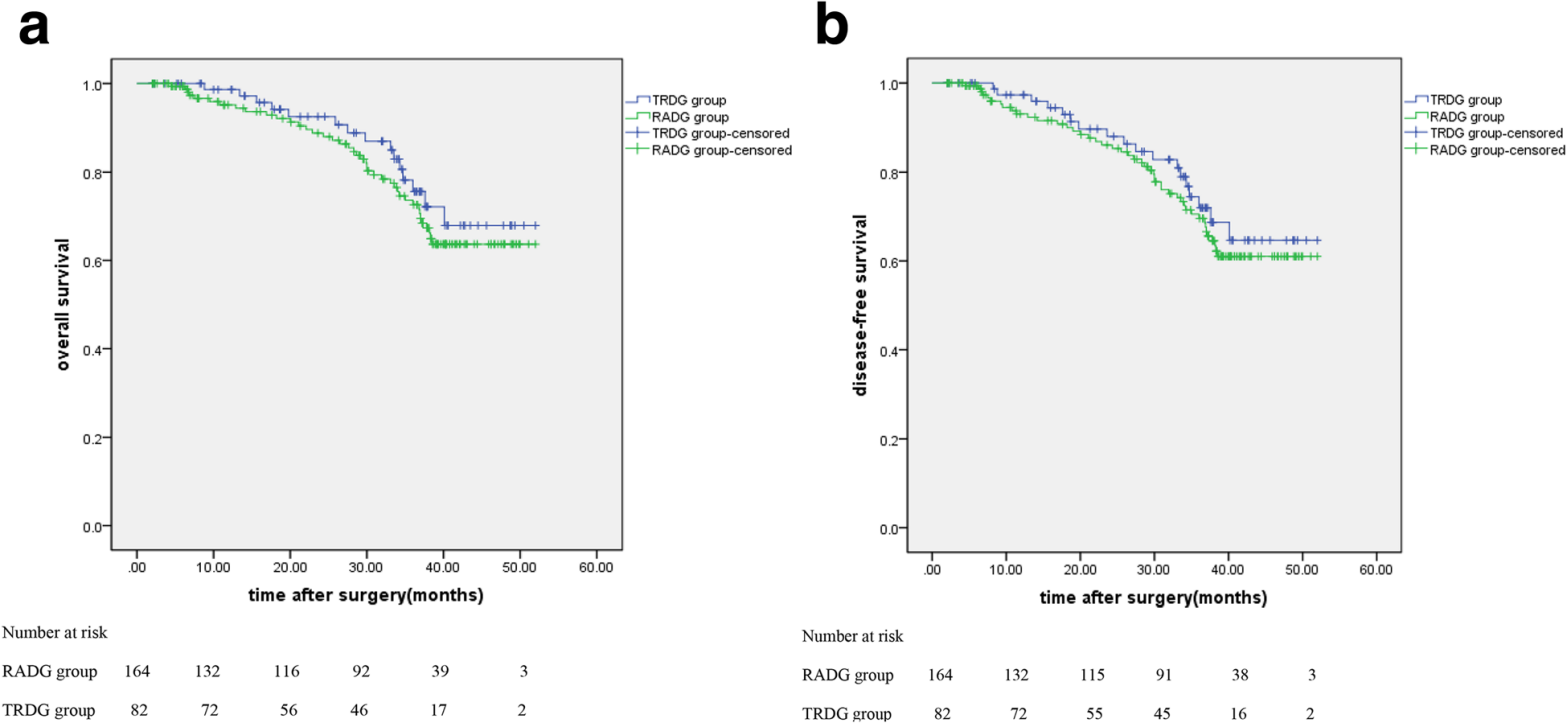
**a** Overall survival. **b** Disease-free survival

Before the end of follow-up, 7 patients (8.5%) in TRDG group and 16 patients (9.8%) in RADG group were censored. Median follow-up time was 33 months ranging from 2 to 52 months for both groups. The 3-year overall survival in TRDG group was 75.6% compared with 72.6% in RADG group (*p* = 0.409) (Fig. 3a). The 3-year disease-free survival in TRDG group is 72.0% compared with 69.6% in RADG group (*p* = 0.482) (Fig. 3b).

## Discussion

Only a few hospitals in mainland China have applied da Vinci robotic-assisted surgical system in use. Moreover, current literatures regarding the role of surgical robot are merely limited to novel technique [13–16] and case reports [17, 18]. The potential of da Vinci robotic-assisted surgical system in this field remains far from being fully understood. Notably, we have noticed that patients undergone TRDG recovered faster in clinical practice, and this study was one of the just few literatures to illustrate the short- and long-outcomes following TRDG and RADG. In terms of these outcomes, our data further verified our hypothesis.

The intra-abdominal bleeding was dramatically decreased in TRDG group compared with RADG group (38.0 ± 18.5 ml vs. 85.6 ± 35.8 ml, *p* = 0.001) which is similar with a recent study [19]. This result was attributed to the superiority that da Vinci robotic-assisted surgical system can filter the physiologic tremor from surgeon and allows surgeon operate in a narrow space so that vascular damage is less. The time to pass flatus were found significantly shorter in TRDG group than that in RADG group (2.7 ± 0.9d vs. 3.8 ± 0.8d, *p* = 0.001). This result could be due to the extracorporeal operation in RADG that brings more stimulation to gastrointestinal tract caused by the dry air. Furthermore, the length of incision in TRDG group was significant decreased in contrast to RADG group (3.3 ± 1.2 cm vs. 6.8 ± 2.5 cm, *p* = 0.001). No extracorporeal operation could account for the advantage of TRDG. So the lessoned trauma on the abdominal wall results in the decreased postoperative activity time in TRDG group than that in RADG group (1.2 ± 0.8d vs. 3.3 ± 0.8d, *p* = 0.001). Besides, patients undergone TRDG had a shorter hospital stay than that in RADG group; this could be explained with the same causes mentioned above. To achieve the safety and oncological outcomes, the time for anastomosis, proximal resection margin, distal resection margin, and number of lymph node dissection were similar in both groups.

Due to the lack of popularity of early screening, a large proportion of diagnosed gastric cancer patients are classified into stages II and III with the expectation to be performed radical gastrectomy. As patients demand surgical incision cosmesis, minimally invasive surgery is gradually recognized. However, the monopoly of surgical robot by Intuitive Surgical Inc. results much more hospitalization cost. Recently, the surgical robot developed by Johnson & Johnson Inc. and Google Inc. will be put on the market, which will cut down the surgical expense to a certain extent.

Surgical stress response plays a key role in surgical outcomes [20]. More surgical trauma results in a higher CRP level [21], and our results (significantly higher CRP and IL-6 levels) were consistent with previous studies [22–24]. The more greater the postoperative inflammatory response is, the more organ disfunction is [21]. Hence, we get that our study confirms that TRDG is ascendant over RADG as a more minimally invasive procedure. However, there exist some limitations in this study; proinflammatory cytokines are secreted by the local injured tissue. Accordingly, these substances are more concentrated in the abdominal cavity than in serum theoretically. A more rigorous detective method should be developed to assess the surgical stress response level in the future research.

Conventionally, most of the digestive tract reconstruction following distal gastrectomy adopts the Billroth I anastomosis prone to result in anastomotic complications, so we adopt the Billroth II anastomosis. The 7-cm incision made at the epigastrium in RADG group makes it easier to misjudge the proximal and distal intestinal tube and cause bleeding by twisting or pulling the intestinal tract. On the contrary, TRDG could maximally minimize this situation. However, we observed no significant differences between the two groups with regard to early complications partially suggesting no difference in short-term outcomes.

Thus far, few literatures with respect to comparing long-term outcomes following TRDG and RADG have been published. Our study earlier proposed the survivorship curve of TRDG and RADG group. We found that there were no significant differences between TRDG group and RADG group either in 3-year overall survival or 3-year disease-free survival supporting considerable oncological safety. Accordingly, we could presume that the prognosis was similar in a way. However, the follow-up time and the sample size were relatively insufficient. To investigate further and obtain more comprehensive data, more follow-up work and data analysis need to be accomplished.

## Conclusion

In conclusion, our current study supports that TRDG is a safe and feasible modus operandi profiting from short- and long-term outcomes compared with RADG. As surgeons are improving their professional skills, TRDG could serve as the standard procedure for distal locally advanced gastric cancer with D2 lymphadenectomy.

## Data Availability

Access to the database can be obtained from the corresponding author on a reasonable request.

## Abbreviations

AGC: Advanced gastric cancer
ASA: American Society Anesthesiologists
BMI: Body mass index
CEA: Carcinoembryonic antigen
CRP: C-reactive protein
DRAS: Da Vinci robotic-assisted surgical system
GC: Gastric cancer
IL-6: Interleukin-6
PCT: Procalcitonin
RADG: Robotic-assisted radical distal gastrectomy
s-CA125: Serum carbohydrate antigen 199
s-CA199: Serum carbohydrate antigen 199
TRDG: Robotic radical distal gastrectomy
WBC: White blood cell
